# Ethyl 5-bromo-3-eth­oxy­carbonyl­amino-1-benzofuran-2-carboxyl­ate

**DOI:** 10.1107/S1600536813002997

**Published:** 2013-02-06

**Authors:** Prashantha Karunakar, V. Krishnamurthy, C. R. Girija, V. Krishna, V. P. Vaidya, A. J. Yamuna

**Affiliations:** a Department of Biotechnology, PES Institute of Technology, BSK III Stg, Bangalore 560 085, India; b Department of Chemistry, SSMRV College, 4th T Block, Jayanagar, Bangalore 560 041, India; c Department of Biotechnology and Bioinformatics, Kuvempu University, Shankarghatta 577 451, India; d Department of Chemistry, Kuvempu University, Jnana Sahyadri, Shankaraghatta 577 451, India

## Abstract

In the title compound, C_14_H_14_BrNO_5_, the ester group is disordered [occupancy ratio 0.52 (2):0.48 (2)]. The major component is nearly coplanar with the benzofuran plane, subtending a dihedral angle of 7.84 (2)°, while the amide group is twisted out of the benzofuran plane making a dihedral angle of 39.69 (2)°. An intra­molecular N—H⋯O hydrogen bond occurs. In the crystal, pairs of weak C—H⋯O hydrogen bonds link the mol­ecules into inversion dimers, which are further linked *via* strong N—H⋯O hydrogen bonds, generating a zigzag chain extending along [100].

## Related literature

For the biological activity of benzofuran derivatives, see: Oter *et al.* (2007 ▶) & Habermann *et al.* (1999 ▶).
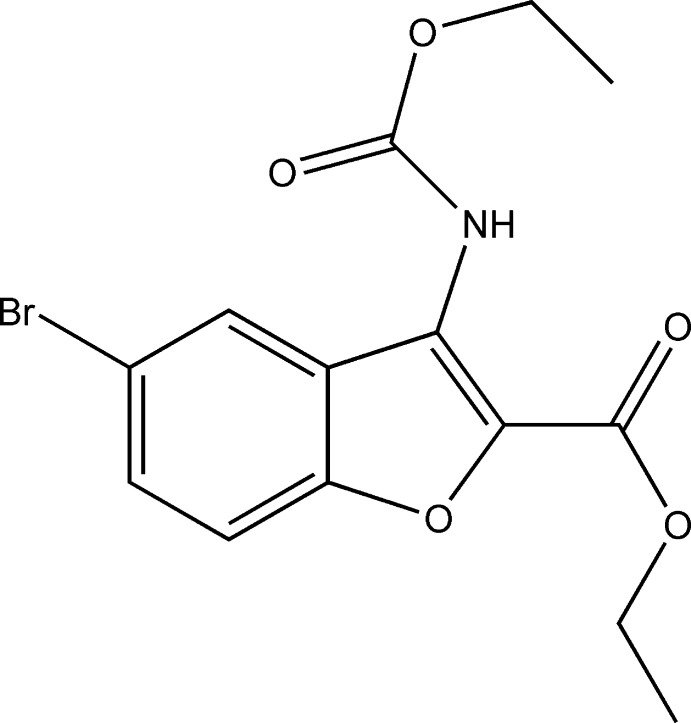


## Experimental

### 

#### Crystal data

C_14_H_14_BrNO_5_
*M* *_r_* = 356.17
Monoclinic, 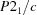

*a* = 14.1960 (7) Å
*b* = 4.8050 (2) Å
*c* = 22.128 (1) Å
β = 90.653 (1)°
*V* = 1509.29 (12) Å^3^
*Z* = 4
Mo *K*α radiation
μ = 2.74 mm^−1^
*T* = 293 K
0.35 × 0.30 × 0.30 mm

#### Data collection

Bruker Kappa APEXII CCD diffractometer
Absorption correction: multi-scan (*SADABS*; Bruker, 2008 ▶) *T*_min_ = 0.426, *T*_max_ = 0.500
14023 measured reflections
2660 independent reflections
1969 reflections with *I* > 2σ(*I*)
*R*_int_ = 0.029

#### Refinement

*R*[*F* ^2^ > 2σ(*F* ^2^)] = 0.038
*wR*(*F* ^2^) = 0.102
*S* = 1.07
2660 reflections
223 parameters
73 restraints
H atoms treated by a mixture of independent and constrained refinement
Δρ_max_ = 0.62 e Å^−3^
Δρ_min_ = −0.60 e Å^−3^

### 

Data collection: *APEX2* (Bruker, 2004 ▶); cell refinement: *APEX2* and *SAINT-Plus* (Bruker, 2004 ▶); data reduction: *SAINT-Plus* and *XPREP* (Bruker, 2004 ▶); program(s) used to solve structure: *SIR92* (Altomare *et al.*, 1993 ▶); program(s) used to refine structure: *SHELXL97* (Sheldrick, 2008 ▶); molecular graphics: *ORTEP-3 for Windows* (Farrugia, 2012 ▶) and Mercury (Macrae *et al.*, 2006 ▶); software used to prepare material for publication: *SHELXL97*.

## Supplementary Material

Crystal structure: contains datablock(s) I, global. DOI: 10.1107/S1600536813002997/hg5285sup1.cif

Structure factors: contains datablock(s) I. DOI: 10.1107/S1600536813002997/hg5285Isup2.hkl

Supplementary material file. DOI: 10.1107/S1600536813002997/hg5285Isup3.cdx

Supplementary material file. DOI: 10.1107/S1600536813002997/hg5285Isup4.cml

Additional supplementary materials:  crystallographic information; 3D view; checkCIF report

## Figures and Tables

**Table 1:** Hydrogen-bond geometry (Å, °)

*D*—H⋯*A*	*D*—H	H⋯*A*	*D*⋯*A*	*D*—H⋯*A*
N1—H1⋯O4	0.89 (1)	2.40 (3)	2.909 (4)	117 (3)
N1—H1⋯O2^i^	0.89 (1)	2.27 (2)	3.021 (4)	142 (3)
C10—H10*B*⋯O4^ii^	0.97 (1)	2.66 (2)	3.427 (4)	135 (3)

Symmetry codes: (i) 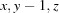; (ii) 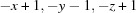.

**Fig. 1.**
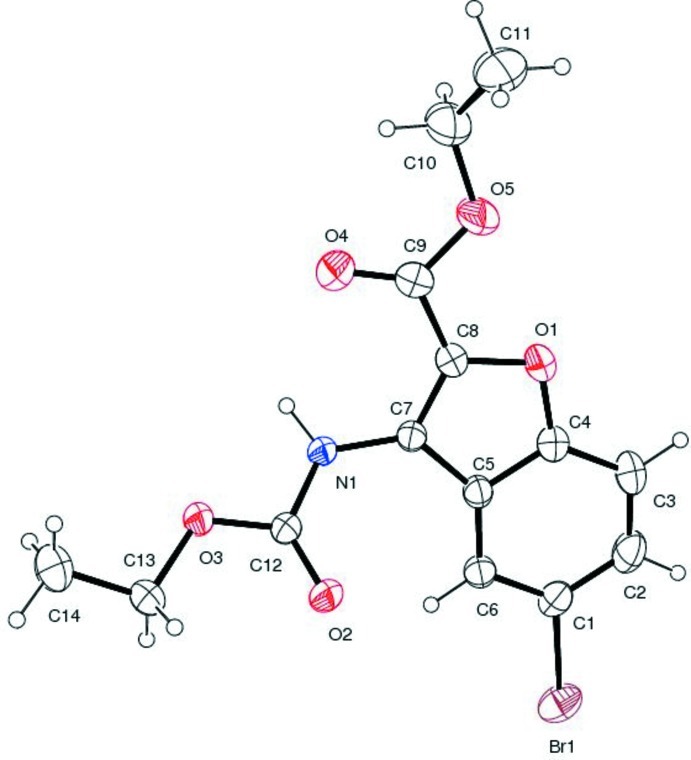
The molecular structure of the title compound, showing the atom-labeling scheme with displacement ellipiods drawn at 50% probability level.

**Fig. 2.**
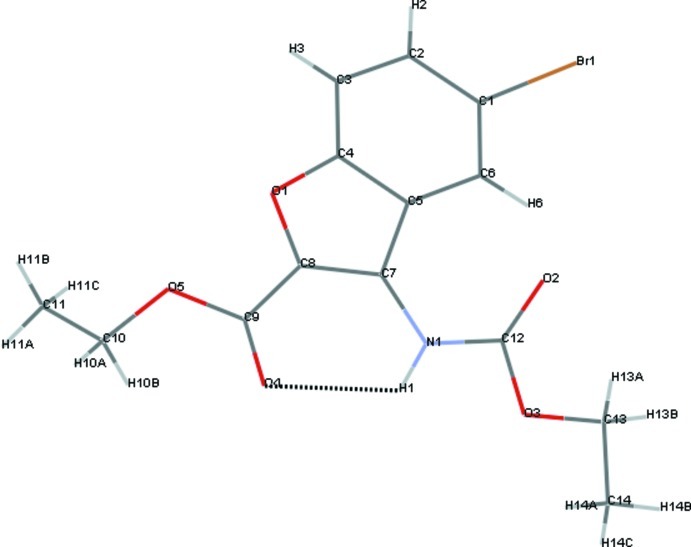
A view of the N-H···O intramolecular interaction (dotted lines) in the crystal structure of the title compound.

**Fig. 3.**
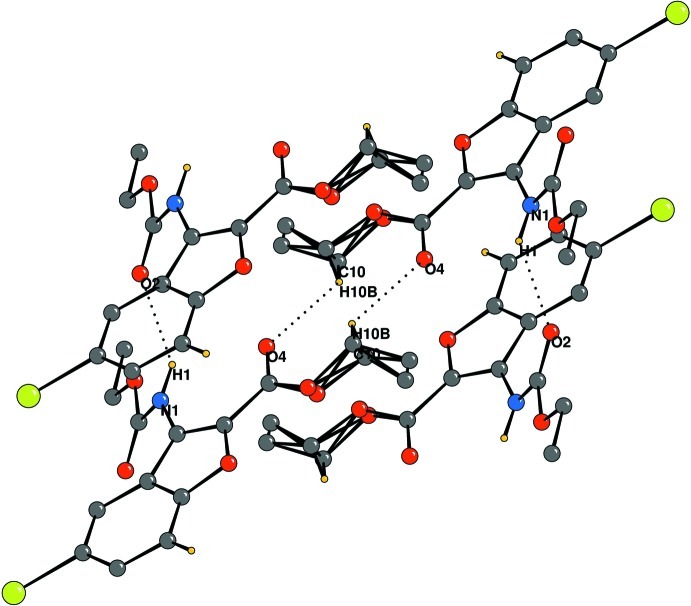
A view of the N—H···O and C—H···O interactions (dotted lines) in the crystal structure of the title compound. H atoms non-participating in hydrogen-bonding were omitted for clarity.

## supplementary crystallographic information

### Comment

Benzofuran derivatives are an important class of heterocyclic compounds that are known to possess variety of biological properties. Substituted benzofurans also find their application in different field such as fluorescent sensor (Oter *et al.*, 2007), antioxidants, brightening agents, a variety of drugs and agriculture (Habermann *et al.*, 1999). In the title compound the ester group is nearly planar with the benzofuran plane with a dihedral angle of 7.84 (2)° while the amide group is twisted out of the benzofuran plane with a dihedral angle of 39.69 (2)°.

The crystal packing is mainly governed by intra- and inter-molecular interactions. An intra-molecular N1—H1···O4 hydrogen bond (Table 1, Figure 2) acting as conformational lock is found between carbonyl O4 and H1 atom of the amide group. The crystal structure is stabilized by inter-molecular interactions N1—H1···O2 (Table 1, Figure 3) resulting in molecular chains along *a* axis. The structure is further stabilised by inter-molecular C10—H10B···O4 interaction (Table 1, figure 3) resulting in the formation of centrosymmetric dimers about the inversion centers.

### Experimental

A mixture of ethyl 3-amino-5-bromobenzofuran-2-carboxylate (2.84 g, 0.01 mol), anhydrous potassium carbonate (2.76 g, 0.02 mol) and ethyl chloroformate (5 ml) in dry benzene (30 ml) was heated under reflux for 8 h. The reaction mixture was filtered and potassium salts were washed with benzene. The filtrate on removal of benzene furnished the desired compound as a yellow solid. It was recrystallized from ethanol (3 g, 84%), m.p. 93–94^o^C.

### Refinement

All carbon-bound hydrogen atoms were placed in calculated positions with C-H distances of 0.95 - 0.99 Å and refined as riding with U_iso_(H) =xU_eq_(C), where x = 1.5 for methyl and x = 1.2 for all other H-atoms. The N-bound H atom positions were determined from difference electron density map and refined freely. One of the ethoxy groups (O5, C10, and C11) was disordered. The disorder was resolved and the final occupancy factors of the components were in the ratio 51:49. The thermal parameters of the atoms of the disordered groups were restrained within an effective standard deviation of 0.02 Å^2.^

### Crystal data

**Table:**

C_14_H_14_BrNO_5_	*F*(000) = 720
*M**_r_* = 356.17	*D*_x_ = 1.567 Mg m^−^^3^
Monoclinic, *P*2_1_/*c*	Melting point: 570.17 K
Hall symbol: -P 2ybc	Mo *K*α radiation, λ = 0.71073 Å
*a* = 14.1960 (7) Å	Cell parameters from 4262 reflections
*b* = 4.8050 (2) Å	θ = 2.3–23.0°
*c* = 22.128 (1) Å	µ = 2.74 mm^−^^1^
β = 90.653 (1)°	*T* = 293 K
*V* = 1509.29 (12) Å^3^	Needle, pale yellow
*Z* = 4	0.35 × 0.30 × 0.30 mm

### Data collection

**Table:**

Bruker Kappa APEXII CCD diffractometer	2660 independent reflections
Radiation source: fine-focus sealed tube	1969 reflections with *I* > 2σ(*I*)
Graphite monochromator	*R*_int_ = 0.029
ω and φ scan	θ_max_ = 25.0°, θ_min_ = 2.3°
Absorption correction: multi-scan (*SADABS*; Bruker, 2008)	*h* = −16→16
*T*_min_ = 0.426, *T*_max_ = 0.500	*k* = −5→3
14023 measured reflections	*l* = −26→26

### Refinement

**Table:**

Refinement on *F*^2^	Primary atom site location: structure-invariant direct methods
Least-squares matrix: full	Secondary atom site location: difference Fourier map
*R*[*F*^2^ > 2σ(*F*^2^)] = 0.038	Hydrogen site location: inferred from neighbouring sites
*wR*(*F*^2^) = 0.102	H atoms treated by a mixture of independent and constrained refinement
*S* = 1.07	*w* = 1/[σ^2^(*F*_o_^2^) + (0.0427*P*)^2^ + 1.449*P*] where *P* = (*F*_o_^2^ + 2*F*_c_^2^)/3
2660 reflections	(Δ/σ)_max_ = 0.002
223 parameters	Δρ_max_ = 0.62 e Å^−^^3^
73 restraints	Δρ_min_ = −0.60 e Å^−^^3^

### Special details

**Table:**

Geometry. All e.s.d.'s (except the e.s.d. in the dihedral angle between two l.s. planes) are estimated using the full covariance matrix. The cell e.s.d.'s are taken into account individually in the estimation of e.s.d.'s in distances, angles and torsion angles; correlations between e.s.d.'s in cell parameters are only used when they are defined by crystal symmetry. An approximate (isotropic) treatment of cell e.s.d.'s is used for estimating e.s.d.'s involving l.s. planes.
Refinement. Refinement of *F*^2^ against ALL reflections. The weighted *R*-factor *wR* and goodness of fit *S* are based on *F*^2^, conventional *R*-factors *R* are based on *F*, with *F* set to zero for negative *F*^2^. The threshold expression of *F*^2^ > σ(*F*^2^) is used only for calculating *R*-factors(gt) *etc*. and is not relevant to the choice of reflections for refinement. *R*-factors based on *F*^2^ are statistically about twice as large as those based on *F*, and *R*- factors based on ALL data will be even larger.

### Fractional atomic coordinates and isotropic or equivalent isotropic displacement parameters (Å^2^)

**Table:**

	*x*	*y*	*z*	*U*_iso_*/*U*_eq_	Occ. (<1)
C1	0.2061 (3)	0.6782 (7)	0.72919 (15)	0.0527 (9)	
C2	0.2889 (3)	0.6822 (9)	0.76285 (17)	0.0676 (11)	
H2	0.2934	0.7955	0.7968	0.081*	
C3	0.3644 (3)	0.5218 (10)	0.74686 (17)	0.0698 (11)	
H3	0.4204	0.5228	0.7691	0.084*	
C4	0.3527 (3)	0.3576 (8)	0.69566 (16)	0.0526 (9)	
C5	0.2708 (2)	0.3532 (7)	0.66123 (14)	0.0439 (8)	
C6	0.1954 (3)	0.5175 (7)	0.67849 (15)	0.0474 (8)	
H6	0.1394	0.5185	0.6563	0.057*	
C7	0.2883 (2)	0.1515 (6)	0.61445 (14)	0.0424 (8)	
C8	0.3765 (2)	0.0536 (7)	0.62420 (16)	0.0508 (9)	
C9	0.4290 (3)	−0.1488 (9)	0.58970 (19)	0.0630 (10)	
C12	0.1724 (2)	0.2280 (7)	0.53506 (14)	0.0432 (8)	
C13	0.0721 (3)	0.2372 (8)	0.44862 (16)	0.0632 (10)	
H13A	0.1022	0.4094	0.4365	0.076*	
H13B	0.0132	0.2823	0.4680	0.076*	
C14	0.0547 (4)	0.0582 (11)	0.39524 (19)	0.0908 (15)	
H14A	0.1136	0.0126	0.3768	0.136*	
H14B	0.0154	0.1555	0.3667	0.136*	
H14C	0.0237	−0.1096	0.4076	0.136*	
N1	0.2276 (2)	0.0576 (6)	0.56904 (13)	0.0486 (7)	
O1	0.41846 (17)	0.1771 (6)	0.67389 (11)	0.0614 (7)	
O2	0.16059 (19)	0.4717 (5)	0.54388 (12)	0.0640 (7)	
O3	0.13305 (17)	0.0840 (5)	0.48991 (10)	0.0550 (6)	
O4	0.3952 (2)	−0.2753 (7)	0.54795 (14)	0.0803 (9)	
Br1	0.10487 (3)	0.90618 (9)	0.754534 (18)	0.0721 (2)	
O5	0.5138 (10)	−0.195 (5)	0.6161 (9)	0.081 (4)	0.52 (2)
C10	0.5720 (9)	−0.418 (4)	0.5900 (9)	0.090 (3)	0.52 (2)
H10A	0.5659	−0.5801	0.6159	0.108*	0.52 (2)
H10B	0.5444	−0.4668	0.5511	0.108*	0.52 (2)
C11	0.6683 (13)	−0.373 (5)	0.5813 (11)	0.114 (5)	0.52 (2)
H11A	0.6970	−0.5406	0.5670	0.171*	0.52 (2)
H11B	0.6974	−0.3182	0.6188	0.171*	0.52 (2)
H11C	0.6765	−0.2275	0.5519	0.171*	0.52 (2)
O5'	0.5217 (11)	−0.153 (6)	0.6020 (9)	0.080 (4)	0.48 (2)
C10'	0.5794 (10)	−0.328 (4)	0.5639 (9)	0.088 (3)	0.48 (2)
H10C	0.5601	−0.5219	0.5656	0.105*	0.48 (2)
H10D	0.5787	−0.2656	0.5223	0.105*	0.48 (2)
C11'	0.6704 (12)	−0.285 (5)	0.5926 (11)	0.109 (5)	0.48 (2)
H11D	0.7170	−0.3945	0.5724	0.163*	0.48 (2)
H11E	0.6675	−0.3409	0.6343	0.163*	0.48 (2)
H11F	0.6870	−0.0919	0.5904	0.163*	0.48 (2)
H1	0.236 (3)	−0.114 (4)	0.5551 (16)	0.066 (12)*	

### Atomic displacement parameters (Å^2^)

**Table:**

	*U*^11^	*U*^22^	*U*^33^	*U*^12^	*U*^13^	*U*^23^
C1	0.068 (2)	0.044 (2)	0.0463 (19)	−0.0026 (17)	0.0021 (18)	−0.0018 (16)
C2	0.086 (3)	0.066 (3)	0.051 (2)	−0.004 (2)	−0.004 (2)	−0.0180 (19)
C3	0.071 (3)	0.080 (3)	0.057 (2)	−0.005 (2)	−0.023 (2)	−0.012 (2)
C4	0.054 (2)	0.053 (2)	0.051 (2)	−0.0006 (18)	−0.0075 (17)	−0.0055 (17)
C5	0.050 (2)	0.0392 (19)	0.0427 (17)	−0.0039 (15)	−0.0093 (15)	0.0021 (14)
C6	0.051 (2)	0.0416 (18)	0.0492 (19)	−0.0017 (16)	−0.0061 (15)	0.0027 (16)
C7	0.049 (2)	0.0352 (18)	0.0432 (18)	0.0007 (15)	−0.0098 (15)	0.0003 (14)
C8	0.048 (2)	0.051 (2)	0.053 (2)	0.0035 (17)	−0.0099 (16)	−0.0051 (17)
C9	0.054 (2)	0.063 (3)	0.071 (3)	0.010 (2)	−0.008 (2)	−0.008 (2)
C12	0.048 (2)	0.0358 (19)	0.0461 (18)	0.0001 (15)	−0.0073 (15)	−0.0025 (15)
C13	0.073 (3)	0.056 (2)	0.060 (2)	0.011 (2)	−0.026 (2)	−0.001 (2)
C14	0.116 (4)	0.096 (4)	0.060 (3)	0.015 (3)	−0.034 (3)	−0.008 (3)
N1	0.0601 (18)	0.0319 (16)	0.0532 (17)	0.0075 (13)	−0.0205 (14)	−0.0060 (13)
O1	0.0541 (15)	0.0690 (17)	0.0606 (15)	0.0056 (13)	−0.0196 (12)	−0.0128 (13)
O2	0.087 (2)	0.0361 (14)	0.0685 (16)	0.0102 (13)	−0.0289 (14)	−0.0078 (12)
O3	0.0703 (16)	0.0411 (13)	0.0530 (14)	0.0065 (12)	−0.0255 (12)	−0.0052 (11)
O4	0.0720 (19)	0.082 (2)	0.087 (2)	0.0160 (16)	−0.0124 (16)	−0.0337 (18)
Br1	0.0910 (4)	0.0599 (3)	0.0658 (3)	0.0063 (2)	0.0203 (2)	−0.0064 (2)
O5	0.051 (4)	0.102 (7)	0.089 (8)	0.024 (4)	−0.008 (4)	−0.026 (6)
C10	0.061 (4)	0.116 (8)	0.093 (7)	0.018 (5)	0.000 (5)	−0.025 (6)
C11	0.103 (8)	0.135 (12)	0.104 (9)	0.043 (7)	0.002 (7)	−0.048 (8)
O5'	0.054 (4)	0.109 (7)	0.078 (7)	0.023 (4)	−0.009 (4)	−0.027 (6)
C10'	0.064 (4)	0.114 (8)	0.086 (7)	0.037 (5)	−0.003 (5)	−0.026 (6)
C11'	0.085 (7)	0.126 (12)	0.116 (9)	0.032 (7)	0.038 (6)	−0.039 (9)

### Geometric parameters (Å, º)

**Table:**

C1—C6	1.369 (5)	C13—O3	1.451 (4)
C1—C2	1.385 (5)	C13—C14	1.480 (5)
C1—Br1	1.897 (4)	C13—H13A	0.9700
C2—C3	1.369 (6)	C13—H13B	0.9700
C2—H2	0.9300	C14—H14A	0.9600
C3—C4	1.389 (5)	C14—H14B	0.9600
C3—H3	0.9300	C14—H14C	0.9600
C4—O1	1.366 (4)	N1—H1	0.886 (10)
C4—C5	1.384 (5)	O5—C10	1.476 (14)
C5—C6	1.386 (5)	C10—C11	1.401 (16)
C5—C7	1.442 (4)	C10—H10A	0.9700
C6—H6	0.9300	C10—H10B	0.9700
C7—C8	1.352 (5)	C11—H11A	0.9600
C7—N1	1.392 (4)	C11—H11B	0.9600
C8—O1	1.379 (4)	C11—H11C	0.9600
C8—C9	1.448 (5)	O5'—C10'	1.451 (14)
C9—O4	1.201 (5)	C10'—C11'	1.448 (16)
C9—O5'	1.342 (15)	C10'—H10C	0.9700
C9—O5	1.350 (14)	C10'—H10D	0.9700
C12—O2	1.199 (4)	C11'—H11D	0.9600
C12—O3	1.333 (4)	C11'—H11E	0.9600
C12—N1	1.354 (4)	C11'—H11F	0.9600
			
C6—C1—C2	122.3 (3)	C13—C14—H14A	109.5
C6—C1—Br1	119.4 (3)	C13—C14—H14B	109.5
C2—C1—Br1	118.3 (3)	H14A—C14—H14B	109.5
C3—C2—C1	121.0 (3)	C13—C14—H14C	109.5
C3—C2—H2	119.5	H14A—C14—H14C	109.5
C1—C2—H2	119.5	H14B—C14—H14C	109.5
C2—C3—C4	116.4 (4)	C12—N1—C7	123.7 (3)
C2—C3—H3	121.8	C12—N1—H1	116 (3)
C4—C3—H3	121.8	C7—N1—H1	118 (2)
O1—C4—C5	111.7 (3)	C4—O1—C8	105.3 (3)
O1—C4—C3	125.0 (3)	C12—O3—C13	116.8 (3)
C5—C4—C3	123.3 (4)	C9—O5—C10	116.7 (14)
C4—C5—C6	119.1 (3)	C11—C10—O5	119.5 (12)
C4—C5—C7	104.7 (3)	C11—C10—H10A	107.5
C6—C5—C7	136.1 (3)	O5—C10—H10A	107.5
C1—C6—C5	117.9 (3)	C11—C10—H10B	107.5
C1—C6—H6	121.0	O5—C10—H10B	107.5
C5—C6—H6	121.0	H10A—C10—H10B	107.0
C8—C7—N1	124.6 (3)	C10—C11—H11A	109.5
C8—C7—C5	106.7 (3)	C10—C11—H11B	109.5
N1—C7—C5	128.7 (3)	H11A—C11—H11B	109.5
C7—C8—O1	111.6 (3)	C10—C11—H11C	109.5
C7—C8—C9	129.1 (3)	H11A—C11—H11C	109.5
O1—C8—C9	119.3 (3)	H11B—C11—H11C	109.5
O4—C9—O5'	122.1 (10)	C9—O5'—C10'	116.8 (15)
O4—C9—O5	126.6 (10)	C11'—C10'—O5'	99.7 (13)
O4—C9—C8	122.8 (3)	C11'—C10'—H10C	111.8
O5'—C9—C8	114.4 (11)	O5'—C10'—H10C	111.8
O5—C9—C8	110.1 (10)	C11'—C10'—H10D	111.8
O2—C12—O3	124.8 (3)	O5'—C10'—H10D	111.8
O2—C12—N1	125.5 (3)	H10C—C10'—H10D	109.6
O3—C12—N1	109.7 (3)	C10'—C11'—H11D	109.5
O3—C13—C14	107.5 (3)	C10'—C11'—H11E	109.5
O3—C13—H13A	110.2	H11D—C11'—H11E	109.5
C14—C13—H13A	110.2	C10'—C11'—H11F	109.5
O3—C13—H13B	110.2	H11D—C11'—H11F	109.5
C14—C13—H13B	110.2	H11E—C11'—H11F	109.5
H13A—C13—H13B	108.5		
			
C6—C1—C2—C3	−0.5 (6)	C7—C8—C9—O5'	164.7 (13)
Br1—C1—C2—C3	179.9 (3)	O1—C8—C9—O5'	−13.8 (13)
C1—C2—C3—C4	−0.1 (6)	C7—C8—C9—O5	−178.0 (12)
C2—C3—C4—O1	−177.6 (4)	O1—C8—C9—O5	3.5 (13)
C2—C3—C4—C5	0.7 (6)	O2—C12—N1—C7	−9.0 (6)
O1—C4—C5—C6	177.7 (3)	O3—C12—N1—C7	170.8 (3)
C3—C4—C5—C6	−0.8 (6)	C8—C7—N1—C12	−138.4 (4)
O1—C4—C5—C7	0.1 (4)	C5—C7—N1—C12	45.2 (5)
C3—C4—C5—C7	−178.3 (4)	C5—C4—O1—C8	−0.3 (4)
C2—C1—C6—C5	0.4 (5)	C3—C4—O1—C8	178.2 (4)
Br1—C1—C6—C5	−180.0 (2)	C7—C8—O1—C4	0.3 (4)
C4—C5—C6—C1	0.2 (5)	C9—C8—O1—C4	179.1 (3)
C7—C5—C6—C1	176.8 (4)	O2—C12—O3—C13	0.4 (5)
C4—C5—C7—C8	0.1 (4)	N1—C12—O3—C13	−179.4 (3)
C6—C5—C7—C8	−176.9 (4)	C14—C13—O3—C12	167.0 (4)
C4—C5—C7—N1	177.0 (3)	O4—C9—O5—C10	2 (2)
C6—C5—C7—N1	0.1 (6)	O5'—C9—O5—C10	−78 (7)
N1—C7—C8—O1	−177.4 (3)	C8—C9—O5—C10	173.7 (11)
C5—C7—C8—O1	−0.3 (4)	C9—O5—C10—C11	136 (2)
N1—C7—C8—C9	4.1 (6)	O4—C9—O5'—C10'	−3 (2)
C5—C7—C8—C9	−178.8 (4)	O5—C9—O5'—C10'	109 (8)
C7—C8—C9—O4	−5.6 (7)	C8—C9—O5'—C10'	−173.0 (13)
O1—C8—C9—O4	175.9 (4)	C9—O5'—C10'—C11'	−178 (2)

### Hydrogen-bond geometry (Å, º)

**Table:**

*D*—H···*A*	*D*—H	H···*A*	*D*···*A*	*D*—H···*A*
N1—H1···O4	0.89 (1)	2.40 (3)	2.909 (4)	117 (3)
N1—H1···O2^i^	0.89 (1)	2.27 (2)	3.021 (4)	142 (3)
C10—H10*B*···O4^ii^	0.97 (1)	2.66 (2)	3.427 (4)	135 (3)

Symmetry codes: (i) *x*, *y*−1, *z*; (ii) −*x*+1, −*y*−1, −*z*+1.
